# Associations with sexual dysfunction among a Canadian cohort with inflammatory bowel disease

**DOI:** 10.1093/jcag/gwae048

**Published:** 2024-11-14

**Authors:** Adam V Weizman, Derek M Nguyen, Laura E Targownik, Jeff Mosko, Natasha Bollegala, Fred Saibil, Vivian Huang, Amanda Selk, Michael Bernstein

**Affiliations:** Division of Gastroenterology, Department of Medicine, Mount Sinai Hospital, University of Toronto, Toronto, Ontario, M5G 1X5, Canada; Division of Gastroenterology, Department of Medicine, Mount Sinai Hospital, University of Toronto, Toronto, Ontario, M5G 1X5, Canada; Division of Gastroenterology, Department of Medicine, Mount Sinai Hospital, University of Toronto, Toronto, Ontario, M5G 1X5, Canada; Division of Gastroenterology, Department of Medicine, St. Michael’s Hospital, University of Toronto, Toronto, Ontario, M5B 1W8, Canada; Division of Gastroenterology, Department of Medicine, Women’s College Hospital, University of Toronto, Toronto, Ontario, M5S1B2, Canada; Division of Gastroenterology, Department of Medicine, Sunnybrook Health Sciences Centre, University of Toronto, Toronto, Ontario, M4N 3M5, Canada; Division of Gastroenterology, Department of Medicine, Mount Sinai Hospital, University of Toronto, Toronto, Ontario, M5G 1X5, Canada; Division of Obstetrics and Gynecology, Department of Obstetrics and Gynecology, Mount Sinai Hospital, Toronto, Ontario, M5G 1X5, Canada; Division of Gastroenterology, Department of Medicine, Sunnybrook Health Sciences Centre, University of Toronto, Toronto, Ontario, M4N 3M5, Canada

**Keywords:** inflammatory bowel disease, ulcerative colitis, Crohn’s disease, sexual dysfunction

## Abstract

**Background:**

Sexual dysfunction is common in individuals with inflammatory bowel disease (IBD). The aim of this study was to better characterize sexual dysfunction and associated factors among a Canadian cohort of IBD patients.

**Methods:**

A cross-sectional survey study was conducted. The primary outcome was sexual dysfunction as measured by the Female Sexual Dysfunction Scale in females and the Male Sexual Dysfunction Scale in males. Analyses were stratified by sex and multiple linear regression was used to identify associations.

**Results:**

In total, 351 respondents completed the survey. Both females and males were impacted by sexual dysfunction (IBD-FSDS 14.1 [± 13.8] and IBD-MSDS 7.2 [± 9.4, respectively]). Depression was common and strongly associated with sexual dysfunction (β coefficient 0.92 [0.13] for men and 0.84 [0.19] for women, *P* <.001).

**Conclusions:**

Sexual dysfunction was common and more impactful in women. Depression was strongly associated with sexual dysfunction. This underscores the need for multidisciplinary care in addressing sexual health among individuals living with IBD.

## Introduction

Sexual dysfunction is defined as difficulty in sexual functioning for a minimum of 6 months and may involve elements of desire, arousal, orgasm, or pain.^1^ It is common in the general population, with some studies demonstrating a prevalence of sexual dysfunction of 43.1% in women and 31% in men.^2^ Inflammatory bowel disease (IBD) is a chronic, relapsing condition of the gastrointestinal tract. These patients are at an increased risk of sexual dysfunction due to disease-related complications, frequent need for surgery and medications, and increased incidence of comorbid anxiety and depression.^3,4^ Previous studies have demonstrated a wide range of reported rates of sexual dysfunction, ranging from 40% to 90% of IBD patients.^5–7^ A recent meta-analysis of 8 studies noted IBD was significantly associated with sexual dysfunction in females (Relative Risk {RR} 1.76 [95% CI, 1.28-2.42], *P* < .001) and males (RR 1.41 [95% CI, 1.09-1.81], *p* = .008).^8^ While there is variation in the reported incidence of sexual dysfunction in IBD patients, the prevalence does appear to be higher than in the general population.

Despite sexual dysfunction being common among individuals living with IBD, there remains a paucity of data studying sexual health in IBD, particularly on what disease-related factors may be more strongly associated with sexual dysfunction. In this study, we aimed to better characterize the burden and predictors of sexual dysfunction among a Canadian cohort of patients with IBD.

## Methods

### Study population

This study was performed across 3 University of Toronto affiliated hospitals (Mount Sinai Hospital, Sunnybrook Health Sciences Centre, and Women’s College Hospital) in Toronto, Ontario, Canada. The hospitals serve a large cohort of adult IBD patients, with the Mount Sinai Centre for IBD site being a large IBD referral centre for complex medical and surgical IBD care. All adult patients over 18 years of age with an established diagnosis of IBD attending ambulatory IBD clinics at participating sites were invited to complete an online, anonymous survey after providing informed consent between August 2022 and December 2023. The only exclusion criterion was the inability to provide informed consent. Patients were recruited to complete the survey through flyers posted at participating clinics as well as through email using the Mount Sinai Hospital IBD Clinic Permission to Contact List which is a research ethics-approved database of 3528 patients at Mount Sinai Hospital who have agreed to be contacted about research studies.

### Study outcomes

**Figure 1.**
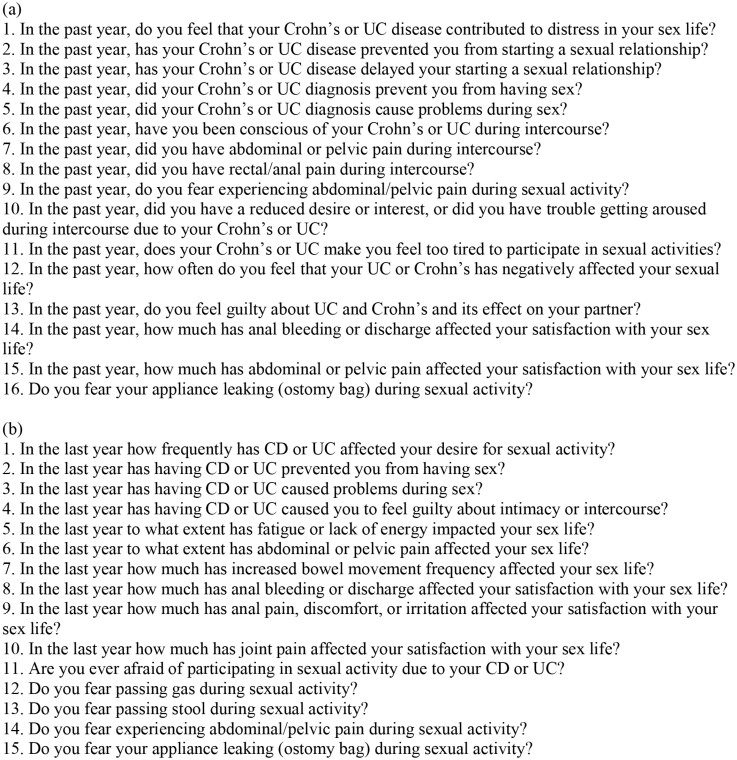
(a) IBD-specific Female Sexual Dysfunction Scale (IBD-FSDS); (b) IBD-specific Male Sexual Dysfunction Scale (IBD-MSDS). 0-4 on a Likert scale [4 = Always or almost always; 3 = Most times (more than half the time); 2 = About half the time; 1 = A few times (less than half the time); and 0 = Never or very rarely].

The primary aim of this cross-sectional survey study was to determine the burden of sexual dysfunction as measured by the IBD-specific Female Sexual Dysfunction Scale (IBD-FSDS)^9^ (Figure 1a) and the IBD-specific Male Sexual Dysfunction Scale (IBD-MSDS)^10^ (Figure 1b). These are both validated screening tools with questions that address the impact of IBD symptoms and psychological factors on sexual dysfunction. Domains within these scores include the impact of symptoms on sex (eg, stool leakage, passing gas, pain, and perianal symptoms), desire, feelings of guilt about sex, fatigue/energy, pain, general and symptom-specific fear/worry, and sex, and stoma related concerns. Responses are on a Likert scale for both, with the IBD-FSDS consisting of 15 items and the IBD-MSDS of 14 items. Each question response is scored from 0 to 4 with a total score then calculated. A higher score is reflective of the greater severity of sexual dysfunction. There is no cutoff value above which sexual dysfunction is defined as present or absent. While other scoring tools have been used in prior studies such as the Female Sexual Function Index, Female Sexual Distress Scale-Revised (FSDS), or Body Image Scale, the IBD-FSDS and IBD-MSDS were chosen for the current study since they have been shown to have strong criterion validity with other scores, strong internal consistency and reliability, are relatively succinct, and focus on psychosexual dysfunction. A third scale that was gender-neutral, the IBD-specific Neutral Sexual Dysfunction Scale, was also included in the survey. Because there were fewer than 5 respondents, it was not included in the analysis due to the low response rate and concerns that individuals could be identified. In addition, measures of trust in physicians were measured using the Trust in Physician Score (TIPS), and comorbid depression was measured using the Patient Health Questionnaire (PHQ-9). These are validated survey tools that have been described elsewhere.^11,12^ Questions on the comfort level in discussing sexual health with their treating gastroenterologist, IBD history, surgery history, medication history, and comorbid disease were also collected.

### Statistical analysis

Statistical analysis included descriptive statistics stratified by sex. Multilevel linear regression was used to identify predictors of sexual dysfunction. Research ethics approval was obtained at all participating sites.

## Results

A total of 351 respondents (162 males and 188 females) completed the survey (Table 1). As the majority of recruitment occurred from the Mount Sinai Hospital IBD Clinic Permission to Contact List, this represented a 10% response rate among those approached. The mean age of respondents was 41.8 (± 14.5) with a mean age of diagnosis of 24.1 (± 11.4). The majority of subjects were married (52%) and identified their sexual orientation as heterosexual (83%). Depression was common and reported in 43% of males and 56% of females (Table 2). Hepatorenal and dermatologic conditions were the most common comorbidities (12% and 16%, respectively). Sixty percent of respondents had Crohn’s disease and 37% ulcerative colitis with the remainder of respondents defining their disease subtype as either IBD-unclassified (IBD-U) or pouch, that is, ileal-pouch anal anastomosis surgery (Table 3). Over half of the patients had a history of IBD surgery. Amongst those with Crohn’s disease, 48% had a history of perianal disease. Approximately two-thirds of patients were on biologic therapy or had been on one previously.

**Table 1.** Demographics of the study cohort.

	Total *n* (%) (*n* = 351)	Male *n* (%) (*n* = 162)	Female *n* (%) (*n* = 188)
Age (SD)	41.8 (14.5)	43.9 (15.3)	40.0 (13.6)
Age at diagnosis (SD)	24.1 (11.4)	25.3 (12.2)	22.9 (10.6)
Gender			
Gender fluid	≤5	≤5	≤5
Man	158 (45)	158 (97)	0
Woman	188 (54)	≤5	186 (99)
Trans man	0	0	0
Trans woman	0	0	0
Prefer not to answer	≤5	0	≤5
Marital status			
Single	101 (29)	46 (28)	55 (29)
Married	182 (52)	90 (56)	92 (49)
Common Law	38 (11)	14 (8)	24 (13)
Divorced	14 (4)	≤5	9 (5)
Separated	8 (2)	≤5	≤5
Widowed	≤5	≤5	≤5
Prefer not to answer	≤5	≤5	≤5
Sexual orientation			
Asexual	7 (2)	≤5	≤5
Bisexual	25 (7)	7 (4)	18 (10)
Gay	≤5	≤5	0
Heterosexual	292 (83)	140 (86)	152 (81)
Lesbian	7 (2)	≤5	6 (3)
Pansexual	≤5	≤5	≤5
Queer	7 (2)	≤5	≤5
Prefer not to answer	≤5	≤5	≤5

**Table 2.** Comorbidities of the study cohort.

	Total (*n* = 351)	Male (*n* = 162)	Female (*n* = 188)
Depression			
Minimal	169 (50)	88 (57)	81 (44)
Mild	94 (28)	40 (26)	53 (29)
Moderate	41 (12)	13 (8)	28 (15)
Moderate severe	21 (6)	9 (6)	12 (6)
Severe	15 (4)	≤5	11 (6)
Comorbidities			
Blood disorder	25 (8)	10 (7)	15 (9)
Cardiovascular	28 (9)	18 (12)	10 (6)
Respiratory	27 (8)	11 (7)	16 (9)
Gastrointestinal	307 (91)	142 (91)	164 (90)
Liver and renal	36 (12)	14 (10)	21 (13)
Neurological	32 (10)	13 (9)	18 (11)
Endocrine	21 (7)	9 (6)	12 (7)
Cancer	24 (8)	12 (8)	12 (7)
Reproductive	24 (8)	7 (5)	17 (10)
Dermatologic	51 (16)	20 (14)	31 (19)
Infectious disease	13 (4)	6 (4)	7 (4)

**Table 3.** Inflammatory bowel disease characteristics and medications of study cohort.

	Total *n* (%) *n* = 351	Male *n* (%) *n* = 162	Female *n* (%) *n* = 188
IBD diagnosis			
Crohn’s disease	208 (60)	101 (63)	106 (58)
Ulcerative colitis	127 (37)	54 (34)	73 (40)
Pouch	6 (2)	≤5	≤5
IBD-unclassified	≤5	≤5	≤5
Disease duration, year (SD)	17.7 (12)	18.5 (11.8)	17.1 (12.1)
IBD surgery	196 (57)	95 (59)	101 (56)
CD complications			
Penetrating	71 (34)	26 (26)	44 (41)
Stricturing	88 (42)	53 (52)	35 (33)
Non-penetrating, non-stricturing	31 (15)	13 (13)	18 (17)
Not sure	18 (9)	9 (9)	9 (8)
CD location			
Ileal	87 (42)	49 (49)	38 (36)
Colonic	32 (15)	16 (16)	15 (14)
Ileocolonic	59 (29)	25 (25)	34 (32)
Not sure	29 (14)	10 (10)	19 (18)
Upper GI Crohn’s disease	41 (20)	16 (16)	24 (23)
Perianal Crohn’s disease	100 (48)	44 (44)	55 (52)
Surgery for perianal CD	52 (25)	24 (24)	28 (26)
UC extent			
Proctitis	18 (14)	6 (10)	12 (16)
Left-sided UC	19 (14)	≤5	14 (19)
Extensive	20 (15)	8 (14)	12 (16)
Not sure	57 (57)	39 (67)	37 (49)
UC severity			
Remission	77 (58)	38 (66)	39 (52)
Mild	35 (26)	10 (17)	25 (33)
Moderate	26 (12)	6 (10)	10 (13)
Severe	≤5	≤5	≤5
IBD medications			
5-aminosalicylates	215 (64)	95 (61)	120 (68)
Steroids	249 (74)	111 (71)	137 (77)
Immunomodulators	154 (46)	73 (47)	81 (46)
Biologics	224 (67)	104 (66)	119 (67)
Infliximab	153 (82)	62 (78)	90 (84)
Adalimumab	87 (56)	37 (53)	50 (59)
Golimumab	≤5	0	≤5
Ustekinumab	51 (37)	25 (38)	25 (35)
Vedolizumab	58 (40)	28 (43)	30 (38)
Tofacitinib	≤5	≤5	≤5

Abbreviation: IBD, inflammatory bowel disease; SD, standard deviation; CD, Crohn’s disease; GI, gastrointestinal; UC, ulcerative colitis.

Mean sexual dysfunction in females as indicated by the IBD-FSDS was 14.1 [± 13.8] and 7.2 [± 9.4] in males as indicated by the IBD-MSDS (Table 4). When comparing the 4 questions in the IBD-FSDS and IBD-MSDS that were the same, females scored higher in all questions, 3 of which were statistically significant (Table 5). Females reported the following impact of IBD on their sexual health at least half the time: 39% felt their IBD negatively affected their sex life; 39% felt guilty about their IBD and how it affected their partner; 34% felt IBD contributed to distress in their sex life; and 35% felt conscious of their IBD during intercourse. Fatigue was a common symptom, affecting 30% of males and 35% of females who reported this impacted their sex life at least half the time.

**Table 4.** Sexual health outcomes and physician trust

	Total *n* (SD) *n* = 351	Male *n* (SD) *n* = 162	Female *n* (SD) *n* = 188
Trust in Physician Score	43.5 (7.5)	44.7 (7.2)	42.4 (7.7)
Female Sexual Dysfunction Survey (mean, SD)	14.1 (13.8)	n/a	14.1 (13.8)
Male Sexual Dysfunction Survey (mean, SD)	7.2 (9.4)	7.2 (9.4)	n/a
Impact fear of leaking ostomy on sexual function			
Never or very rarely	29 (54)	17 (63)	12 (44)
A few times (less than half the time)	≤5	≤5	≤5
About half the time	≤5	≤5	≤5
Most times (more than half the time)	7 (13)	≤5	≤5
Always or almost always	12 (22)	≤5	7 (25)
Discussion sexual health with a physician	48 (15)	21 (14)	27 (16)
The physician initiated a discussion on sexual health	19 (6)	7 (5)	12 (7)
Comfortable discussing sexual health with a physician	256 (79)	135 (87)	120 (71)^a^
Physician’s gender affects comfort in discussing sexual health	71 (22)	19 (12)	51 (30)^a^
Virtual care increases comfort in discussing sexual health	88 (27)	35 (23)	52 (31)

^a^ *P*≤.001.

**Table 5.** Comparison of males and females among common elements of the sexual dysfunction surveys.

Affected at least half the time	Sex		*P*-value	Adjusted odds ratio (aOR) Female vs Male (95% CI)^a^
	Males *n* (SD) *n* = 154	Females *n* (SD) *n* = 173		
In the past/last year, did your Crohn’s or UC diagnosis prevent you from having sex?	19 (12)	47 (27)	.001	2.30 (1.15-4.62)
In the past/last year, did your Crohn’s or UC diagnosis cause problems during sex?	16 (10)	30 (18)	.06	1.59 (0.75-3.38)
In the past/last year, how much has anal bleeding or discharge affected your satisfaction with your sex life?	11 (7)	24 (14)	.04	1.39 (0.56-3.45)
In the past/last year, did you fear experiencing abdominal/pelvic pain during sexual activity?	11 (7)	42 (25)	<.001	3.52 (1.61-7.70)

Abbreviation: IBD, inflammatory bowel disease; SD, standard deviation; UC, ulcerative colitis.

^a^Adjusted for age, marital status, IBD diagnosis, history of IBD surgery, depression (PHQ9 score), and Trust in Physician Score.

Regression analysis results are shown in Table 6. Depression, as indicated by PHQ9 scores, was associated with sexual dysfunction in both males and females (β coefficient 0.92 [0.13] and 0.84 [0.19], *P* < .001, respectively). Prior IBD surgery was significantly associated with sexual dysfunction in females (4.09 [2.03] *P* = .046) but not in males, although a history of a pouch in males was a significant association (23.8 [3.33] *P* < .001).

**Table 6.** Regression analyses adjusting for clinical and sociodemographic factors.

	Males		Females	
	β coefficient (SE)	*P*-value	β coefficient (SE)	*P*-value
Age	−0.05 (0.04)	.18	0.09 (0.11)	.39
Married	1.44 (1.54)	.35	3.22 (2.32)	.17
IBD diagnosis (CD vs UC)				
Crohn’s disease	Ref		Ref	
Ulcerative colitis	−0.59 (1.47)	.69	−3.73 (2.07)	.07
Pouch	23.8 (3.33)	<.001	14.3 (12.8)	.27
IBD-unclassified	0.34 (2.77)	.90	−3.43 (3.73)	.36
Prior IBD surgery	0.08 (1.56)	.96	4.09 (2.03)	.046
PHQ9 Score	0.92 (0.13)	<.001	0.84 (0.19)	<.001
Trust in Physician Score	−0.20 (0.10)	.06	−0.14 (0.14)	.32
Discussion with MD	1.83(2.24)	.42	1.84 (2.65)	.49
MD Initiating Discussion	8.26 (6.56)	.21	0.91 (3.77)	.81
Comfort with MD	−1.10 (2.08)	.60	0.25 (2.54)	.92
Gender of MD	−0.71 (2.28)	.76	7.28 (2.45)	.004
Virtual visit with MD	0.89 (1.66)	.59	2.84 (2.13)	.19

Abbreviation: IBD, inflammatory bowel disease; SE, standard error; CD, Crohn’s disease; UC, ulcerative colitis; MD, medical doctor.

Overall, 15% of respondents had discussed sexual health with their physician and among these, only 6% of these discussions were initiated by the physician. There was no difference in trust in physicians among men and women as measured by the TIPS (44.7 and 42.4, respectively). However, male patients were more comfortable discussing sexual health with their physician (87% vs 71%, *P* < .001) and female patients more often felt their physician’s gender affected their comfort in discussing sexual health (30% vs 12%, *P* < .001). Almost a third of responders indicated they would be more comfortable addressing sexual health over virtual care as compared to in-person.

## Discussion

Sexual dysfunction is a well-known complication of individuals living with IBD. There is significant heterogeneity in studies reporting the prevalence of sexual dysfunction in males and females with IBD. Previous studies have demonstrated a wide range in reported rates of sexual dysfunction, ranging from 40% to 90% of females with IBD and 15%-25% of men with IBD.^5–8^ A recent systematic review and meta-analysis of 13 studies noted a pooled global prevalence of sexual dysfunction in 61.4% [95% CI, 52.8%-70.1%] of women with IBD.^13^ Consistent across all studies is the finding that sexual dysfunction is more prevalent among women with IBD as compared to men.^14^

In the current study, we present the largest Canadian cohort to date evaluating the impact of IBD on sexual dysfunction. As with previous studies, our results demonstrated that sexual dysfunction was frequently reported by patients and that females were more impacted as compared to males, as indicated by significantly higher sexual dysfunction scores in females for common elements among the IBD-FSDS and IBD-MSDS. A variety of contributors to the consistently higher incidence of sexual dysfunction in females have been described. Females with IBD have been demonstrated to have significantly more impairment of sexual desire, arousal, lubrication, orgasm, and pain as compared to women without IBD.^13^ Females with IBD are also more likely to report a reduction in intercourse frequency due to fatigue, abdominal pain, and fear of incontinence during intercourse.^15^ IBD can cause perianal disease and vulvovaginal disorders, which may impact body image more in women and may also be more likely to cause pain with intercourse.^14^ Comorbid depression and anxiety have been demonstrated to be more common in females with IBD and are among the strongest associations of sexual dysfunction in individuals with IBD.^9,10,16,17^

We noted depression to be common among our cohort with over half of patients reporting some depression. A recent study from New Zealand identified that severe depression was associated with sexual dysfunction in females (OR [Odds Ratio] 5.77; 95% CI, 1.59-20.94) and anxiety was associated with erectile dysfunction in males (OR 15.62; 95% CI, 1.74-140.23).^18^ A meta-analysis of 13 studies noted depression to be significantly associated with sexual dysfunction in females.^13^ Depression was an important predictor of sexual dysfunction in a male cohort of IBD patients (OR 3.1 95% CI, 1.5-6.2), particularly for sexual satisfaction with less impact specifically on erectile or orgasmic function.^19^ There are a number of potential explanations for this strong and consistent association. Comorbid depression has been associated with less interest in sex and has been associated with impairment in all domains of sexual health in both males and females.^20–22^ Moreover, a more severe IBD course has been associated with a higher incidence of depression, and many patients in our cohort were followed at a large IBD referral centre and were, therefore, more likely to have severe and complex IBD. We also noted that over 50% of patients noted more than minimal symptoms of depression. This underscores the high prevalence of depression in our cohort, which could be a reflection of a cohort with a more severe disease course. In addition, while depression can negatively impact sexual function, sexual dysfunction can, in turn, lead to worsened psychological health, and worsening depressive symptoms.^18^ Treatment of depression has been associated with improvement of sexual dysfunction.^23^ The strong association between depression and sexual dysfunction highlights the need for a multidisciplinary approach that includes mental health professionals who can address sexual health among individuals with IBD.

Very few patients in our cohort discussed sexual dysfunction with their physician and when discussed, this topic was usually patient-initiated. This finding is in agreement with a survey of 480 adults with IBD that found only 5.7% received information on sexual health from their providers.^20^ This finding underscores there still remains a stigma in discussing sexual health in those with IBD. Patients are often uncomfortable raising the issue and providers may feel they lack adequate training in addressing this issue and therefore do not raise it. Previous studies have identified several barriers in addressing sexual dysfunction including patient discomfort, insufficient time, lack of a holistic approach to address issues on sexuality, and previous poor experiences with other providers if this had been discussed.^20^ Despite this, previous studies have shown that 40%-50% of patients expected or wanted a discussion on their sexual issues with their gastroenterologist.^20^ Barriers addressing mental health issues in IBD have been frequently identified as a shortcoming in IBD care, and the high prevalence of comorbid depression speaks to the need for multidisciplinary care with attention to both mental and physical health. Further studies could explore whether proper treatment of depression can mitigate the negative impacts of IBD on sexual health. Interestingly, we noted that female patients indicated they would be more comfortable addressing sexual health using virtual care. The use of virtual care has increased exponentially since the COVID pandemic. Perhaps the comfort of home allows some of the above barriers to be overcome; opportunities to leverage virtual care to better address sexual dysfunction need to be explored. Table 7 highlights potential strategies providers could use to start addressing sexual dysfunction among individuals living with IBD.

**Table 7.** Strategies to address sexual dysfunction.

Incorporate questions around sexual health in routine IBD follow-up Consider asking about sexual symptoms and libido when screening for symptoms suggestive of disease activity Screen for depression and anxiety with referral to appropriate mental health professional when identified Multidisciplinary team approach to IBD care

Abbreviation: IBD, inflammatory bowel disease.

The current study has several limitations. The responses were based on patient self-report and therefore subject to recall and response bias. Some domains, for example, a history of surgery, were self-defined and it is conceivable there may have been variability in reporting what was considered a surgery. An examination under anaesthesia (EUA) or incision and drainage of a perianal abscess may be considered surgery by some respondents but not by others. This may explain the high rates of surgery in our cohort, although the majority of subjects were followed at a tertiary IBD centre and may have had more severe diseases and were more likely to require surgery. This could potentially impact the generalizability of the results. Due to the sensitive nature of the topic, some patients may have been uncomfortable in completing the surveys thereby creating a selection bias. Secondly, the instruments used to measure and diagnose sexual dysfunction presumed gender to be binary, whereas some patients may not identify with these classifications, thereby impacting their ability to use the IBD-MSDS and IBD-FSDS. We attempted to address this by allowing subjects to report their gender with more expansive definitions. It should be noted that no respondent in our survey self-identified as transgender. This suggests that the majority of respondents felt either the IBD-MSDS or IBD-FSDS adequately captured their responses. In addition, the bulk of the patients in the study were followed at Mount Sinai Hospital, which follows complex IBD patients. Therefore, the results may not be applicable to the general IBD population. However, our results are in agreement with previous studies that have used a more general cohort of patients. Moreover, over half of patients with ulcerative colitis indicated they were in remission suggesting a significant subset of respondents felt their disease was under control. We did not specifically measure disease activity at the time of survey completion. As disease activity and active IBD symptoms play a major role in sexual well-being and dysfunction, this is an important limitation to highlight. The inclusion of disease activity scores into the survey could be considered in follow-up studies. Lastly, this was a cross-sectional study design, and we, therefore, cannot make any conclusions on a causal relationship between associations noted with sexual health.

In conclusion, we have noted sexual dysfunction in both male and female patients with IBD. Depression was common and a strong predictor of poor sexual health. Whether this independently impacts sexual health or is a marker of comorbid severe IBD needs to be further studied along with the impact of treating depression on sexual health. The results of the study underscore the importance of multidisciplinary care in these patients with particular attention to mental health. Sexual health should be discussed more frequently during routine ambulatory IBD care, and more studies addressing the barriers as to why this is not taking place are needed. Virtual care may be a promising opportunity to perhaps increase comfort with the discussion of sexual health.

## Supplementary Material

gwae048_suppl_Supplementary_Material

## Data Availability

The data from this study are available upon request.
